# Titling the NP: Challenges and Misconceptions

**Published:** 2014-11-01

**Authors:** Catherine Bishop

**Affiliations:** Dr. Bishop is an oncology nurse practitioner at Johns Hopkins Kimmel Cancer Center/Sibley Hospital in Washington, DC.

Nurse practitioners (NPs) have been practicing for nearly 50 years (American Association of Nurse Practitioners [AANP], 2014). So why is there still confusion over who we are and what we do? Why do we consistently get questions from patients such as "Are you my nurse?" or "Are you my doctor?" or "Are you the assistant?" And perhaps even more confounding, why is there still confusion about the NP role within the nursing and medical communities?

There are likely many reasons for this confusion, which seems to be fairly consistent across institutions and private practices. Inconsistency in both messaging and scope of practice across states is a potential factor that comes to mind. While revising scope of practice is a mandate taken up by individual states and driven by individual NP organizations, it takes great effort, persistence, and time to navigate the legislative process. Currently, NPs have full practice authority in 19 states and the District of Columbia (AANP, 2014). For NPs in the remaining states, there is a varying degree of restrictive language with respect to their practice authority, creating a myriad of issues for patients and NPs alike.

In 2010, the Institute of Medicine (IOM) published a report entitled, "The Future of Nursing: Leading Change, Advancing Health" (IOM, 2010). This report put forth 5 recommendations, number 1 of which was to remove scope-of-practice barriers. Many NPs face this hindrance today, which negatively impacts the patients in their care. Removing scope-of-practice barriers would legally allow advanced practice registered nurses (APRNs) to practice to the full extent of their education and training. Nearly in tandem, the National Council of State Boards of Nursing (NCSBN) established the APRN Consensus Model, which mirrors the philosophy of the IOM report in standardizing advanced practice nursing. The APRN Consensus Model was developed by 48 APRN nursing organizations that are committed to creating nationwide standards to unite all NPs (and all other advanced practice nurses) in their licensure, accreditation, certification, and education (NCSBN, 2010).

## THE POWER OF MARKETING

The APRN Consensus Model also creates standards for all NPs, which may help to clear up the existing confusion and perhaps once and for all come up with an answer to "Who are we?" But marketing ourselves with a consistent message is paramount in achieving the goal and definitively answering the question.

At my institution, we created an NP council to work on "getting the word out" about who we are and what we do. We began by taking small steps toward educating our administrators, nursing colleagues, physician colleagues, and patients about our education, our scope of practice, and our roles within the health-care team. We worked with the institution’s marketing and public relations departments to create informational articles for publications that are distributed to the local health-care community as well as to the local general community. With the help of the public relations team, we took a group photo to accompany the article and came up with a short description of what it means to be an NP and what role we play in health care. Our clinical biographies will be published in a separate publication. As a further step, we are working on getting the institution’s website to include profile links for all of the NPs, including photos and biographies.

## LISTING CREDENTIALS

Another simple way to further consistent messaging is to standardize the way we display our credentials after our names. Peruse articles written by NPs and likely you’ll see a variety of approaches to listing credentials. It’s not hard to imagine how this could be confusing to coworkers and patients alike.

Along with many of my colleagues, I was taught that placing the highest degree earned directly after your name and the role in which you practice right after that is the most appropriate and accepted method of listing your credentials. I look to my PhD colleagues, who almost consistently display their credentials in this manner: Mary Smith, PhD, RN; or John Jones, PhD, NP. Certification is important and can be added at the end.

However, confusion reigns when NPs continue to use RN after their names. You either practice as an RN or an NP. In every state and the District of Columbia, NPs have a distinct and separate scope of practice from RNs. This distinction is what makes us unique and allows us to practice beyond the scope of an RN. If we want to clarify who we are to our medical, nursing, and patient communities, we must start by uniting and presenting a clear message.

Physician assistants, pharmacists, and other advanced practice colleagues—you have done a good job of creating consistency with your credentials. Bravo to you!

To achieve the goal of accurately defining ourselves (and not allowing others to define us) as NPs let us consider standing together and displaying our credentials in a consistent manner: highest earned degree first, then role, and finally certification. This may begin the job of educating our colleagues and helping our patients truly understand who we are.

## YOUR PERSPECTIVE

What kind of experiences have you had in trying to explain what it is that you do? What is the feedback from your institution? What are your ideas for taking steps to define ourselves in our changing health-care field? The editors of JADPRO would love to hear your feedback. Please write to editor@advancedpractitioner.com to share your unique perspective!
